# Association between obesity and short- and medium-term mortality in critically ill patients with atrial fibrillation: a retrospective cohort study

**DOI:** 10.1186/s12872-023-03179-x

**Published:** 2023-03-23

**Authors:** Duo Yang, Shujun Ye, Kaihong Zhang, Zhiliang Huang, Longsheng Zhang

**Affiliations:** 1 Department of Anesthesiology, Jieyang People’s Hospital, No. 107 Tianfu Road, Rongcheng District, Jieyang, Guangdong Province 522000 China; 2 Guangdong Medical University, No. 2 Wenming East Road, Xiashan District, Zhanjiang, Guangdong Province 524023 China

**Keywords:** Obesity, Atrial fibrillation, Critically ill patients, Mortality, Body mass index

## Abstract

**Background:**

There has been controversy about how obesity affects the clinical prognosis for patients with atrial fibrillation (AF), and the relationship between obesity and outcomes in critically ill patients with AF remains unclear. The purpose of this study was to explore the association between obesity and short- and medium-term mortality in critically ill patients with AF.

**Methods:**

The Medical Information Mart for Intensive Care-IV (MIMIC-IV) database was used to conduct a retrospective cohort analysis on 9282 critically ill patients with AF. Patients were categorized into four groups based on their body mass index (BMI) values: underweight, normal-weight, overweight, and obese. The outcomes of this study were 30-day, 90-day, and 1-year all-cause mortality. Cox proportional-hazards models and restricted cubic spline analyses were performed to investigate the association between BMI and mortality.

**Results:**

For 30-day mortality, after adjustment for all confounding factors, the hazard ratio (HR) with 95% confidence interval (CI) for the underweight, overweight, and obese categories were 1.58 (1.21, 2.07), 0.82 (0.72, 0.93), and 0.79 (0.68, 0.91), respectively, compared to the normal-weight category. Using multivariable-adjusted restricted cubic spline analysis, an “L-shaped” correlation was observed between BMI and 30-day mortality. For each 1 kg/m^2^ increase in BMI when BMI was less than 30 kg/m^2^, the risk of 30-day mortality decreased by 6.4% (HR, 95% CI: 0.936 [0.918, 0.954]; *P* < 0.001); however, this relationship was not present when BMI was greater than or equal to 30 kg/m^2^. Similar results were observed for 90-day and 1-year mortality.

**Conclusions:**

There was a nonlinear relationship between BMI and all-cause mortality among critically ill patients with AF. All-cause mortality and the BMI were negatively correlated when the BMI was less than 30 kg/m^2^.

**Supplementary Information:**

The online version contains supplementary material available at 10.1186/s12872-023-03179-x.

## Background

As the most prevalent kind of persistent arrhythmia, atrial fibrillation (AF) is associated with severe morbidity and mortality, placing a heavy burden on patients and healthcare systems worldwide [1]. It is estimated that the number of people suffering from AF will reach 72 million by 2050 [2]. AF is characterized by a severe disturbance of atrial electrical activity, resulting in ineffective atrial contractions [3]. It is a significant cause of heart failure, stroke, and all-cause mortality [4]. AF is also the most prevalent arrhythmia in patients admitted to the intensive care unit (ICU) [5]. ICU patients with AF experience worse clinical outcomes than patients without AF [6]. Reducing the adverse prognoses of critically ill patients with AF has always been an important clinical goal [7]. Identifying prognostic factors among critically ill patients with AF is crucial for clinicians to make wiser treatment strategies and improve clinical outcomes.

Over the past 40 years, obesity has shown a clear upward trend in both developed and developing countries and has become a global epidemic [8]. Obesity was found to be an independent risk factor for the occurrence, recurrence, and progression of AF in previous studies [9–11]. Interestingly, some studies showed that overweight and obesity were positively associated with better outcomes among patients with AF [12, 13]. The “obesity paradox” has been used to describe this counter-intuitive phenomenon. In fact, the obesity paradox has been reported to exist in various cardiovascular diseases, including heart failure [14], hypertension, and coronary artery disease [15], as well as various non-cardiovascular diseases, including chronic liver disease [16] and chronic obstructive pulmonary disease [17]. Nonetheless, there is conflicting evidence regarding how obesity affects clinical outcomes in AF patients [18]. In a prospective cohort study, overweight and obesity were indicated to be associated with adverse outcomes in AF patients [19]. In addition, most of the previous studies have focused on the association of obesity with long-term outcomes in AF patients. To date, in critically ill patients with AF, the relationship between obesity and outcomes remains unclear. Therefore, we aimed to use real-world data from the Medical Information Mart for Intensive Care-IV (MIMIC-IV) database to evaluate the relationship between obesity and all-cause mortality for critically ill patients with AF.

## Methods

### Data source

The data for this retrospective cohort study was collected from the MIMIC-IV database (version 2.1), a publicly accessible database containing the clinical information on over 73,000 ICU stays at Beth Israel Deaconess Medical Center (BIDMC) from 2008 to 2019. The use of the database has been approved by the Massachusetts Institute of Technology and the BIDMC institutional review boards. Duo Yang, one of the authors, has obtained access to the database (certification number: 48247201). Information in this database was anonymous, and informed consent from the participants was waived. This study adhered to the Declaration of Helsinki and the guidelines for Strengthening the Reporting of Observational Studies in Epidemiology.

### Study Population

The inclusion criteria of our study were adult critically ill patients (age ≥ 18 years) diagnosed with atrial fibrillation according to the ninth or tenth revision of the International Classification of Diseases (ICD) code. ICD-9 code 427.31, ICD-10 codes I48.0, I48.1x, I48.2x, and I48.91 were used to identify the patients [20, 21]. Patients were excluded if key data (weight on the first day admitted to the ICU, height, or follow-up information) were missing. Additionally, patients with a length of ICU stay less than 24 h were also excluded. Finally, for patients who had multiple ICU stays, only the data of the first ICU admission were analyzed in this study.

### Data extraction

Navicat Premium 12 software and Structure Query Language were utilized to extract the following information from the MIMIC-IV database: (1) weight on the first day of ICU stay and height; (2) demographics: age, sex, and race; (3) comorbidities: hypertension, diabetes, congestive heart failure (CHF), peripheral vascular disease (PVD), cerebrovascular disease, chronic pulmonary disease (CPD), renal disease, liver disease, malignancy, sepsis, acute kidney injury (AKI), acute heart failure (AHF), and stroke; (4) disease severity scores: sequential organ failure assessment (SOFA), simplified acute physiology score II (SAPS II), and Charlson comorbidity index (CCI); (5) the first laboratory parameter and vital sign value after ICU admission: hemoglobin, white blood cell (WBC), platelet, red cell distribution width (RDW), anion gap, blood urea nitrogen (BUN), creatinine, glucose, heart rate (HR), mean blood pressure (MBP), respiratory rate (RR), and saturation of pulse oximetry (SpO_2_); (6) treatment information: medication administration during hospitalization (antiplatelet agents, anticoagulant agents, antiarrhythmic agents, and vasopressors), mechanical ventilation (MV), and renal replacement treatment (RRT).

### Exposure variable and study endpoints

Body mass index (BMI), the most widely used anthropometric measure of adiposity, was the exposure variable in this study. The formula for calculating BMI is weight (in kilograms)/height (in meters)^2^. According to the World Health Organization BMI classifications, participants were categorized into four groups: underweight (BMI < 18.5 kg/m^2^), normal-weight (BMI: 18.5 to < 25 kg/m^2^), overweight (BMI: 25 to < 30 kg/m^2^), and obese (BMI ≥ 30 kg/m^2^). The endpoints of our study were 30-day, 90-day, and 1-year all-cause mortality after ICU admission. 30-day and 90-day mortality were referred to as short-term, whereas 1-year mortality as medium-term mortality.

### Statistical analysis

Participant characteristics were analyzed based on the BMI categories. Categorical variables were described as numbers (percentages) and chi-square test was used for comparison between groups. Continuous variables were described as mean ± standard deviation (SD) for normal distributions or median and interquartile range (IQR) for skewed distributions. One-way analysis of variance or Kruskal–Wallis *H*-test was applied to evaluate the statistical difference of continuous variables.

Survival analyses were visualized using Kaplan–Meier curves and compared with a log-rank test. Cox proportional-hazards models were performed to evaluate the hazard ratio (HR) and 95% confidence interval (CI) for the relationship between BMI and mortality. For multivariable Cox analyses, covariates were chosen according to previous findings and clinical constraints. We also adjusted for covariates that had *P* values less than 0.1 in single-factor Cox analysis or that, when added to the model, changed the matched odds ratio by at least 10% [22]. The variance inflation factor (VIF) was utilized to identify the multicollinearity of the covariates in the fully adjusted models, and the VIFs of all covariates cannot be greater than 5. In the minimally adjusted model (Model 1), adjusted variables included age, sex, and race. In the fully adjusted model (Model 2), we further adjusted for hypertension, diabetes, CHF, PVD, cerebrovascular disease, CPD, renal disease, liver disease, malignancy, sepsis, AKI, AHF, stroke, SOFA, SAPS II, CCI, hemoglobin, WBC, platelet, RDW, anion gap, BUN, creatinine, glucose, HR, MBP, RR, SpO_2_, antiplatelet agents, anticoagulant agents, antiarrhythmic agents, MV, RRT, and vasopressors.

Multivariable-adjusted restricted cubic spline analyses were conducted to evaluate the possible nonlinear association between BMI and all-cause mortality. If a nonlinear association was detected, an optimal inflection point for BMI was determined, and a two-segment linear regression model was used to assess the threshold effect of the BMI on all-cause mortality. The log-likelihood ratio test was performed to compare the one-line linear model with a two-segment linear regression model. Subgroup analyses were conducted by age, sex, and comorbidities that may influence the relationship between BMI and mortality. The likelihood ratio tests were used to examine interactions between subgroups.

We simply replaced the missing data using the mean and median because no more than 2% of each covariate in the data was missing. Sensitivity analyses were performed to evaluate the robustness of our results. We analyzed whether the association between BMI and mortality would change after excluding patients with missing data. Furthermore, to investigate study outcomes distinctively in different levels of obesity, patients with BMI ≥ 30 kg/m^2^ were divided into two groups: obese (BMI: 30 to < 40 kg/m^2^) and morbidly obese (BMI ≥ 40 kg/m^2^).

Data analyses were conducted using the statistical software package R. version 4.0.5 (R Foundation, Vienna, Austria) and Free Statistics software version 1.7.1. Differences with a two-sided *P* < 0.05 were considered to be statistically significant.

## Results

### Selection of participants

**Fig. 1:**
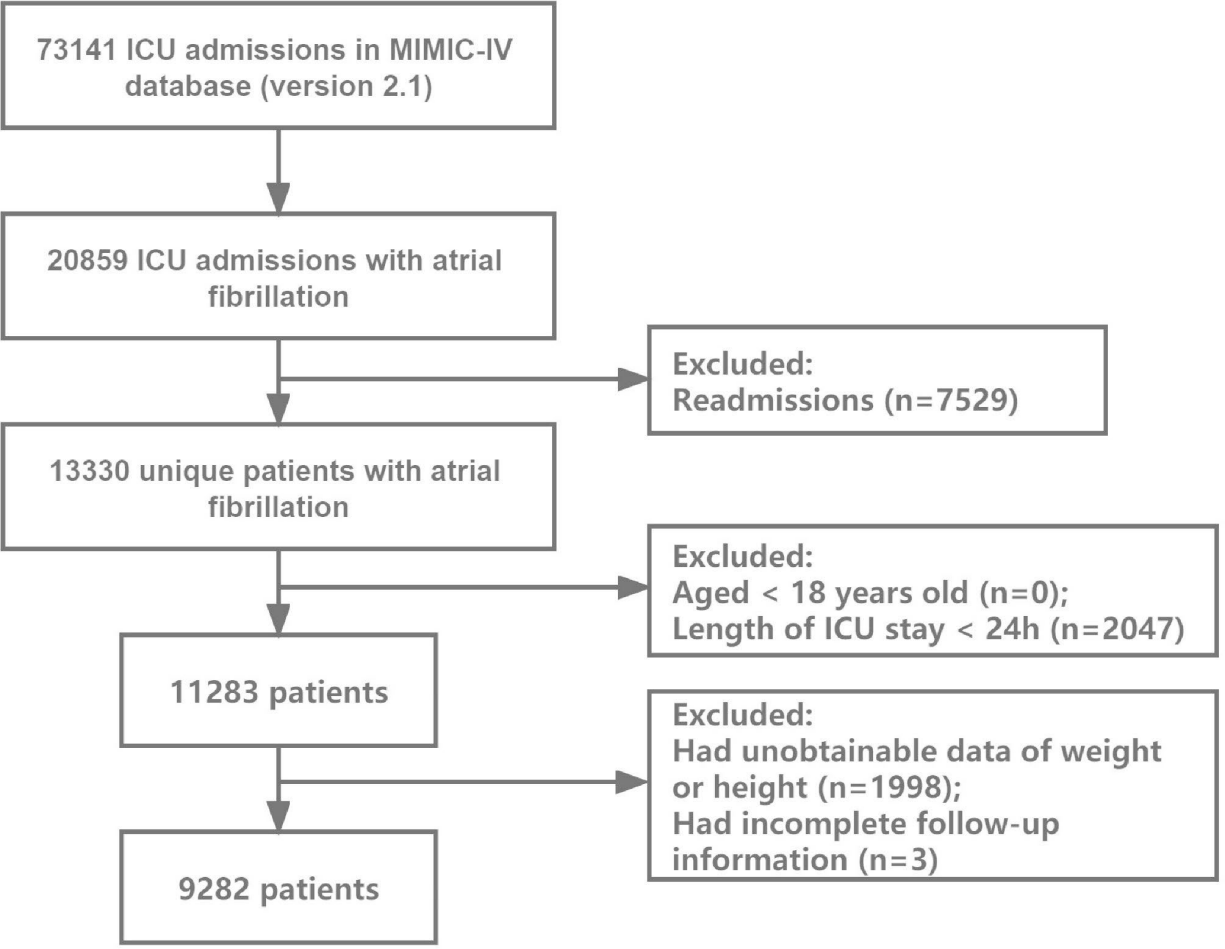
Flowchart of the study cohort. Abbreviations: ICU, intensive care unit; MIMIC-IV, Medical Information Mart for Intensive Care-IV

Of the 73,141 ICU admissions, 13,330 patients with atrial fibrillation were identified. A flowchart of this study is presented in Fig. 1. The cohort for the final analysis included 9,282 participants.

### Baseline characteristics of participants

The baseline characteristics of the participants stratified by BMI values are shown in Table 1. According to the BMI values, 199, 2357, 3114, and 3612 patients belonged to the underweight, normal-weight, overweight, and obese categories, respectively. The mean age of all patients was 74.2 ± 11.5 years, with males accounting for 60.5% of the population. The median SOFA score of all participants was 5.0 (IQR [3.0, 8.0]); SAPS II scores averaged 40.5 ± 13.0; and CCI scores averaged 6.6 ± 2.5. Patients with a higher BMI were younger, and men accounted for a larger percentage of those patients. Compared with normal-weight patients, overweight and obese patients had a higher prevalence of hypertension and diabetes, whereas underweight patients presented more history of cerebrovascular disease, CPD, and malignancy. Overall, patients with a higher BMI had lower 30-day, 90-day, and 1-year all-cause mortality. Interestingly, patients with a higher BMI were more likely to receive relevant treatments, including antiplatelet agents, vasopressors, MV, and RRT.

**Table 1:** Baseline characteristics of the study population according to BMI category

Variables	Total	BMI category (kg/m^2^)				*P*-Value
		Underweight	Normal-weight	Overweight	Obese	
		< 18.5	≥ 18.5, < 25	≥ 25, < 30	≥ 30	
N	9282	199	2357	3114	3612	
Age (years)	74.2 ± 11.5	77.3 ± 13.2	78.0 ± 11.5	75.1 ± 11.0	70.8 ± 11.0	< 0.001
Sex (male)	5612 (60.5)	76 (38.2)	1261 (53.5)	2068 (66.4)	2207 (61.1)	< 0.001
Race (White)	6905 (74.4)	146 (73.4)	1731 (73.4)	2323 (74.6)	2705 (74.9)	0.622
**Comorbidities**						
Hypertension	4266 (46.0)	72 (36.2)	1024 (43.4)	1438 (46.2)	1732 (48)	< 0.001
Diabetes	2938 (31.7)	26 (13.1)	482 (20.4)	896 (28.8)	1534 (42.5)	< 0.001
CHF	4193 (45.2)	88 (44.2)	1072 (45.5)	1347 (43.3)	1686 (46.7)	0.044
PVD	1494 (16.1)	28 (14.1)	402 (17.1)	514 (16.5)	550 (15.2)	0.202
Cerebrovascular disease	1495 (16.1)	41 (20.6)	422 (17.9)	523 (16.8)	509 (14.1)	< 0.001
CPD	2756 (29.7)	89 (44.7)	695 (29.5)	844 (27.1)	1128 (31.2)	< 0.001
Renal disease	2497 (26.9)	41 (20.6)	594 (25.2)	813 (26.1)	1049 (29)	< 0.001
Liver disease	734 (7.9)	15 (7.5)	175 (7.4)	224 (7.2)	320 (8.9)	0.058
Malignancy	1146 (12.3)	39 (19.6)	338 (14.3)	414 (13.3)	355 (9.8)	< 0.001
Sepsis	5463 (58.9)	103 (51.8)	1372 (58.2)	1812 (58.2)	2176 (60.2)	0.046
AKI	7471 (80.5)	134 (67.3)	1742 (73.9)	2472 (79.4)	3123 (86.5)	< 0.001
AHF	2129 (22.9)	43 (21.6)	530 (22.5)	683 (21.9)	873 (24.2)	0.148
Stroke	828 (8.9)	27 (13.6)	241 (10.2)	291 (9.3)	269 (7.4)	< 0.001
**Disease severity scores**						
SOFA	5.0 (3.0, 8.0)	5.0 (3.0, 7.0)	5.0 (3.0, 7.0)	5.0 (3.0, 8.0)	5.0 (3.0, 8.0)	< 0.001
SAPS II	40.5 ± 13.0	41.7 ± 13.2	41.5 ± 12.5	40.5 ± 13.1	39.8 ± 13.2	< 0.001
CCI	6.6 ± 2.5	6.9 ± 2.5	6.7 ± 2.4	6.6 ± 2.6	6.5 ± 2.6	0.013
**Laboratory parameters**						
Hemoglobin (g/dL)	10.6 ± 2.3	10.4 ± 2.3	10.6 ± 2.3	10.6 ± 2.4	10.7 ± 2.3	0.008
WBC (×10^9^/L)	11.0 (7.9, 15.1)	10.6 (7.0, 14.2)	10.5 (7.3, 14.5)	10.8 (7.8, 15.0)	11.6 (8.4, 15.8)	< 0.001
Platelet (×10^9^/L)	179.0 (132.0, 242.0)	221.0 (155.0, 318.0)	184.0 (129.0, 250.0)	173.0 (127.0, 236.0)	180.0 (136.8, 237.0)	< 0.001
RDW (%)	15.0 ± 2.2	15.5 ± 2.5	15.0 ± 2.3	14.9 ± 2.2	15.0 ± 2.1	< 0.001
Anion gap (mmol/L)	14.7 ± 4.6	15.1 ± 4.5	14.9 ± 4.6	14.6 ± 4.7	14.7 ± 4.5	0.079
BUN (mg/dL)	21.0 (15.0, 33.0)	22.0 (16.0, 37.0)	22.0 (15.0, 32.0)	21.0 (15.0, 33.0)	21.0 (16.0, 35.0)	0.116
Creatinine (mg/dL)	1.0 (0.8, 1.5)	0.9 (0.6, 1.4)	1.0 (0.7, 1.4)	1.0 (0.8, 1.5)	1.1 (0.8, 1.6)	< 0.001
Glucose (mg/dL)	126.0 (106.0, 156.0)	116.0 (98.5, 138.0)	122.0 (102.0, 150.0)	126.0 (106.0, 154.0)	131.0 (110.0, 165.0)	< 0.001
**Vital signs**						
HR (beats/minute)	88.1 ± 21.2	92.0 ± 22.0	88.0 ± 21.2	87.8 ± 21.4	88.1 ± 20.9	0.056
MBP (mmHg)	81.6 ± 18.1	84.2 ± 21.7	82.0 ± 17.5	81.8 ± 18.1	81.1 ± 18.1	0.034
RR (beats/minute)	18.6 ± 5.8	19.6 ± 6.0	18.9 ± 6.0	18.6 ± 6.0	18.4 ± 5.6	< 0.001
SpO_2_ (%)	97.1 ± 4.1	96.6 ± 5.1	97.1 ± 4.4	97.2 ± 4.0	97.1 ± 4.0	0.085
**Therapy**						
Antiplatelet agents	3288 (35.4)	53 (26.6)	775 (32.9)	1133 (36.4)	1327 (36.7)	< 0.001
Anticoagulant agents	4348 (46.8)	103 (51.8)	1093 (46.4)	1415 (45.4)	1737 (48.1)	0.077
Antiarrhythmic agents	4421 (47.6)	98 (49.2)	1094 (46.4)	1472 (47.3)	1757 (48.6)	0.354
MV	3586 (38.6)	55 (27.6)	813 (34.5)	1154 (37.1)	1564 (43.3)	< 0.001
RRT	657 (7.1)	11 (5.5)	141 (6)	198 (6.4)	307 (8.5)	< 0.001
Vasopressors	5111 (55.1)	93 (46.7)	1226 (52)	1719 (55.2)	2073 (57.4)	< 0.001
**Outcomes**						
30-day all-cause mortality	1405 (15.1)	66 (33.2)	451 (19.1)	458 (14.7)	430 (11.9)	< 0.001
90-day all-cause mortality	1912 (20.6)	82 (41.2)	642 (27.2)	617 (19.8)	571 (15.8)	< 0.001
1-year all-cause mortality	2714 (29.2)	109 (54.8)	891 (37.8)	872 (28)	842 (23.3)	< 0.001

Note: Variables are presented as mean ± SD, median (IQR), or N (%)

Abbreviations: BMI, body mass index; CHF, congestive heart failure; PVD, peripheral vascular disease; CPD, chronic pulmonary disease; AKI, acute kidney injury; AHF, acute heart failure; SOFA, sequential organ failure assessment; SAPS II, simplified acute physiology score II; CCI, Charlson comorbidity index; WBC, white blood cell; RDW, red cell distribution width; BUN, blood urea nitrogen; HR, heart rate; MBP, mean blood pressure; RR, respiratory rate; SpO_2_, saturation of pulse oximetry; MV, mechanical ventilation; RRT, renal replacement therapy

### Effects of BMI on Mortality

**Fig. 2:**
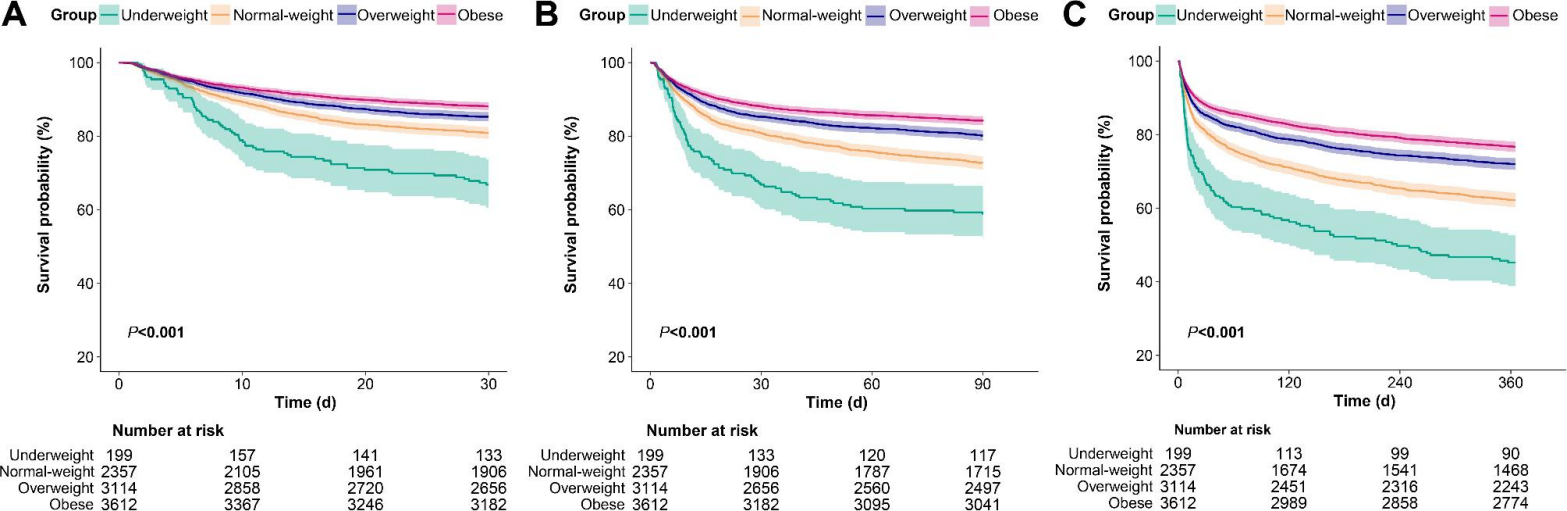
Kaplan–Meier curves of 30-day (A), 90-day (B), and 1-year (C) all-cause mortality by BMI categories. Abbreviations: BMI, body mass index

The Kaplan–Meier curves for 30-day, 90-day, and 1-year survival are presented in Fig. 2 (all *P* values for log-rank were less than 0.001). Both single-factor and multivariable Cox proportional risk models were conducted to evaluate the association between the BMI and all-cause mortality in critically ill patients with AF. The results of the single-factor Cox regression analysis of covariates and all-cause mortality are presented in **Supplementary Table 1**. In Table 2, we show both the crude and adjusted models. When BMI was considered a categorical variable (the four BMI categories), we used the normal-weight category as a reference group for comparison with other groups. For 30-day mortality, in the fully adjusted model (Model 2), the HR with 95% CI for the underweight, overweight, and obese categories were 1.58 (1.21, 2.07), 0.82 (0.72, 0.93), and 0.79 (0.68, 0.91), respectively, compared to the normal-weight category (all *P* values < 0.05). For 90-day mortality, in Model 2, the HR with 95% CI for the underweight, overweight, and obese categories were 1.46 (1.15, 1.85), 0.76 (0.68, 0.85), and 0.7 (0.62, 0.79), respectively, compared to the normal-weight category (all *P* values < 0.05). For 1-year mortality, in Model 2, the HR with 95% CI for the underweight, overweight, and obese categories were 1.45 (1.19, 1.78), 0.76 (0.69, 0.83), and 0.7 (0.63, 0.77), respectively, compared to the normal-weight category (all *P* values < 0.05).

**Table 2:** Relationship between BMI and all-cause mortality among critically ill patients with atrial fibrillation

Variables	Crude model		Model 1		Model 2	
	h (95% CI)	*P*-value	HR (95% CI)	*P*-value	HR (95% CI)	*P*-value
30-day all-cause mortality						
BMI category						
Underweight	1.89 (1.46, 2.44)	< 0.001	1.92 (1.48, 2.48)	< 0.001	1.58 (1.21, 2.07)	0.001
Normal-weight	1.00 (Reference)		1.00 (Reference)		1.00 (Reference)	
Overweight	0.75 (0.66, 0.85)	< 0.001	0.84 (0.74, 0.96)	< 0.001	0.82 (0.72, 0.93)	0.003
Obese	0.6 (0.52, 0.68)	< 0.001	0.78 (0.68, 0.9)	< 0.001	0.79 (0.68, 0.91)	0.001
*P* for trend		< 0.001		< 0.001		< 0.001
90-day all-cause mortality						
BMI category						
Underweight	1.7 (1.35, 2.14)	< 0.001	1.73 (1.38, 2.18)	< 0.001	1.46 (1.15, 1.85)	0.002
Normal-weight	1.00 (Reference)		1.00 (Reference)		1.00 (Reference)	
Overweight	0.7 (0.63, 0.78)	< 0.001	0.79 (0.7, 0.88)	< 0.001	0.76 (0.68, 0.85)	< 0.001
Obese	0.55 (0.49, 0.61)	< 0.001	0.71 (0.63, 0.8)	< 0.001	0.7 (0.62, 0.79)	< 0.001
*P* for trend		< 0.001		< 0.001		< 0.001
1-year all-cause mortality						
BMI category						
Underweight	1.69 (1.39, 2.06)	< 0.001	1.71 (1.4, 2.09)	< 0.001	1.45 (1.19, 1.78)	< 0.001
Normal-weight	1.00 (Reference)		1.00 (Reference)		1.00 (Reference)	
Overweight	0.7 (0.63, 0.76)	< 0.001	0.78 (0.71, 0.85)	< 0.001	0.76 (0.69, 0.83)	< 0.001
Obese	0.56 (0.51, 0.62)	< 0.001	0.72 (0.65, 0.79)	< 0.001	0.7 (0.63, 0.77)	< 0.001
*P* for trend		< 0.001		< 0.001		< 0.001

Note: Crude model was adjusted for none; Model 1 was adjusted for age, sex and race; Model 2 was further adjusted (from Model 1) for hypertension, diabetes, CHF, PVD, cerebrovascular disease, CPD, renal disease, liver disease, malignancy, sepsis, AKI, AHF, stroke, SOFA, SAPS II, CCI, hemoglobin, WBC, platelet, RDW, anion gap, BUN, creatinine, glucose, HR, MBP, RR, SpO_2_, antiplatelet agents, anticoagulant agents, antiarrhythmic agents, MV, RRT, and vasopressors

Abbreviations: BMI, body mass index; HR, hazard ratio; CI, confidence interval; CHF, congestive heart failure; PVD, peripheral vascular disease; CPD, chronic pulmonary disease; AKI, acute kidney injury; AHF, acute heart failure; SOFA, sequential organ failure assessment; SAPS II, simplified acute physiology score II; CCI, Charlson comorbidity index; WBC, white blood cell; RDW, red cell distribution width; BUN, blood urea nitrogen; HR, heart rate; MBP, mean blood pressure; RR, respiratory rate; SpO_2_, saturation of pulse oximetry; MV, mechanical ventilation; RRT, renal replacement therapy

**Fig. 3:**
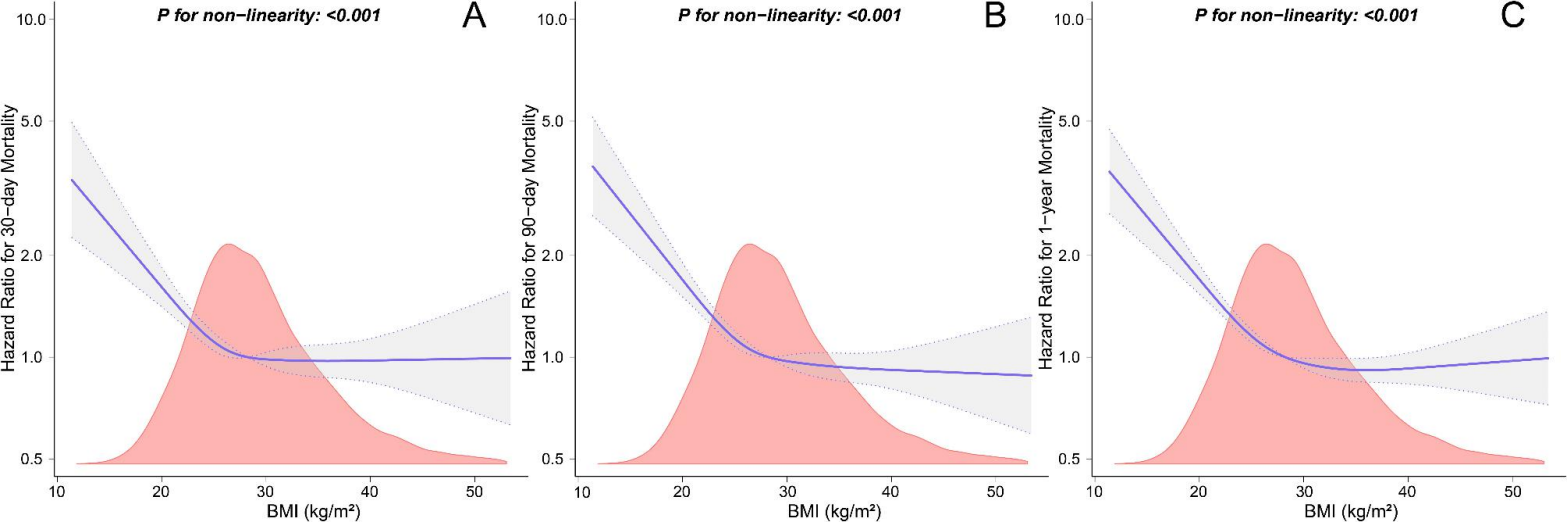
Multivariable-adjusted restricted cubic spline analyses of relationship between BMI and 30-day (A), 90-day (B), and 1-year (C) all-cause mortality. The upper limit of the BMI is restricted to 99th. The purple lines represent the estimated risk of all-cause mortality, and the gray bands represent the point-by-point 95% CI adjusted for covariates. HRs were adjusted for age, sex, race, hypertension, diabetes, CHF, PVD, cerebrovascular disease, CPD, renal disease, liver disease, malignancy, sepsis, AKI, AHF, stroke, SOFA, SAPS II, CCI, hemoglobin, WBC, platelet, RDW, anion gap, BUN, creatinine, glucose, HR, MBP, RR, SpO_2_, antiplatelet agents, anticoagulant agents, antiarrhythmic agents, MV, RRT, and vasopressors. Abbreviations: BMI, body mass index; HR, hazard ratio; CI, confidence interval; CHF, congestive heart failure; PVD, peripheral vascular disease; CPD, chronic pulmonary disease; AKI, acute kidney injury; AHF, acute heart failure; SOFA, sequential organ failure assessment; SAPS II, simplified acute physiology score II; CCI, Charlson comorbidity index; WBC, white blood cell; RDW, red cell distribution width; BUN, blood urea nitrogen; HR, heart rate; MBP, mean blood pressure; RR, respiratory rate; SpO_2_, saturation of pulse oximetry; MV, mechanical ventilation; RRT, renal replacement therapy.

We also treated BMI as a continuous variable. Using multivariable-adjusted restricted cubic spline analysis, an “L-shaped” correlation between BMI and 30-day mortality was evident (*P* for non-linearity < 0.001), and a similar correlation was found for 90-day and 1-year all-cause mortality (Fig. 3). Combining the graphical interpretation with clinical utility, an optimal inflection point for BMI was determined to be 30 kg/m^2^. For each 1 kg/m^2^ increase in BMI when BMI was less than 30 kg/m^2^, the risk of 30-day mortality decreased by 6.4% (HR, 95% CI: 0.936 [0.918, 0.954]; *P* < 0.001). However, this relationship was not present when BMI was greater than or equal to 30 kg/m^2^ (HR, 95% CI: 0.996 [0.974, 1.017]; *P* = 0.689). Similarly, for each 1 kg/m^2^ increase in BMI when BMI was less than 30 kg/m^2^, the risk of 90-day and 1-year mortality decreased by 6.9% (HR, 95% CI: 0.931 [0.916, 0.947]; *P* < 0.001) and 6.8% (HR, 95% CI: 0.932 [0.919, 0.946]; *P* < 0.001), respectively. This relationship was also not present when BMI was greater than or equal to 30 kg/m^2^ for 90-day (HR, 95% CI: 0.992 [0.974, 1.011]; *P* = 0.412) and 1-year mortality (HR, 95% CI: 0.996 [0.981, 1.011]; *P* = 0.588) (Table 3).

**Table 3:** Threshold effect analysis of the relationship between BMI and all-cause mortality

Threshold of BMI		30-day all-cause mortality			90-day all-cause mortality			1-year all-cause mortality	
		HR (95%CI)	*P*-value		HR (95%CI)	*P*-value		HR (95%CI)	*P*-value
< 30 kg/m^2^		0.936 (0.918, 0.954)	< 0.001		0.931 (0.916, 0.947)	< 0.001		0.932 (0.919, 0.946)	< 0.001
≥ 30 kg/m^2^		0.996 (0.974, 1.017)	0.689		0.992 (0.974, 1.011)	0.412		0.996 (0.981, 1.011)	0.588
Likelihood ratio test			< 0.001			< 0.001			< 0.001

Note: HRs were adjusted for age, sex, race, hypertension, diabetes, CHF, PVD, cerebrovascular disease, CPD, renal disease, liver disease, malignancy, sepsis, AKI, AHF, stroke, SOFA, SAPS II, CCI, hemoglobin, WBC, platelet, RDW, anion gap, BUN, creatinine, glucose, HR, MBP, RR, SpO_2_, antiplatelet agents, anticoagulant agents, antiarrhythmic agents, MV, RRT, and vasopressors

Abbreviations: BMI, body mass index; HR, hazard ratio; CI, confidence interval; CHF, congestive heart failure; PVD, peripheral vascular disease; CPD, chronic pulmonary disease; AKI, acute kidney injury; AHF, acute heart failure; SOFA, sequential organ failure assessment; SAPS II, simplified acute physiology score II; CCI, Charlson comorbidity index; WBC, white blood cell; RDW, red cell distribution width; BUN, blood urea nitrogen; HR, heart rate; MBP, mean blood pressure; RR, respiratory rate; SpO_2_, saturation of pulse oximetry; MV, mechanical ventilation; RRT, renal replacement therapy

### Subgroup analyses and sensitivity analyses

Further subgroup analyses were performed on 5670 patients with BMI < 30 kg/m^2^ to verify the consistency of the correlation between mortality and BMI. The interaction tests were not statistically significant for most stratification variables (**Supplementary Fig. 1**). Hypertension, AHF, and AKI modified the association between 30-day mortality and BMI. Hypertension, sepsis, AHF, and AKI modified the association between 90-day mortality and BMI. Sex, hypertension, and AHF modified the association between 1-year mortality and BMI. However, in all of these interactive stratifications, BMI acted as a protective factor.

In the sensitivity analyses, results remained robust after excluding participants with incomplete data of covariates (**Supplementary Tables 2, Supplementary Tables 3**, and **Supplementary Fig. 2**). When patients with BMI ≥ 30 kg/m^2^ were divided into obese and morbidly obese group, the results of multivariable Cox regression analyses indicated that morbidly obese patients still had a survival advantage, compared to normal-weight patients (**Supplementary Table 4**). For 30-day mortality, in the fully adjusted model, the HR with 95% CI for the morbidly obese category was 0.77 (0.6, 0.98), compared to the normal-weight category (*P =* 0.034). Similar results were found for 90-day and 1-year mortality.

## Discussion

This study focused on the association between BMI and short- and medium-term mortality among critically ill patients with AF. We found that underweight patients had higher 30-day, 90-day, and 1-year all-cause mortality compared with normal-weight patients, even after adjusting for essential confounders, including important disease severity scores (SOFA, SAPS II, and CCI). Conversely, overweight and obese patients had a relatively low risk of death. Furthermore, when BMI was considered a continuous variable, we observed a curvilinear association between BMI and mortality among critically ill patients with AF. The dose-response effect of BMI on all-cause mortality was significantly different when it was below or above the threshold of 30 kg/m^2^, showing that a threshold effect was present. Stratified analyses and sensitivity analyses suggested the robustness of our results. This information will contribute to an in-depth understanding of the relationship between the BMI and all-cause mortality of critically ill patients with AF, thereby strengthening the physician’s ability to risk-stratify patients.

The obesity paradox has recently received a lot of attention in various diseases. In previous studies, overweight and obese patients with AF were reported to have a long-term survival advantage compared to normal-weight patients [12, 13, 23]. Obesity has also been shown to have a protective effect in other critically ill patient populations, including sepsis [24], AKI [25], and coronary care unit patients [26]. Most of the participants in these studies were from Western populations, and our study cohort also included only a very small number of Asians. Differences in obesity standards between Asian and Western populations may lead to ethnic differences in findings on the obesity paradox. Nevertheless, in a Chinese study, overweight AF patients had lower all-cause mortality and cardiovascular mortality compared with normal-weight and underweight patients [27]. In another study conducted in Japan, being overweight was associated with a reduced risk of all-cause death among AF patients [28]. Our findings in critically ill patients with AF were in accordance with these studies. However, a prospective cohort study showed that overweight and obesity are risk factors for adverse clinical outcomes among AF patients [19]. In this research, the study population was not critically ill patients, and the study outcome was a composite endpoint of ischemic stroke, thromboembolism, or death. Therefore, the results do not represent the relationship between obesity and mortality in critically ill patients with AF. Another study conducted by Wang et al. showed that overweight or obesity may be a risk factor for poor prognoses in AF patients [29]. But their study population was also not exactly the same as ours. Although there are a lot of studies on the obesity paradox, the detailed mechanisms remain poorly understood.

One reason for this phenomenon may relate to the higher metabolic reserve in patients with obesity. As reported, AF patients with a higher BMI are better able to endure the increased catabolic stress associated with disease development [30]. A catabolic state with increased energy expenditure is an important feature in the early stages of critical illness [31]. Patients with obesity have a better ability to supply more substrate synthesis energy to fulfill the increased demands during such a period because of their large lipid reserves [32]. In addition to increasing available energy, adipose tissue can play a role in critical illness by improving insulin sensitivity, reducing dyslipidemia, and enhancing thermoregulation. Although the underlying mechanism is not fully understood, white adipose tissue browning may be an important pathway for these effects [33].

Other protective effects provided by adipose tissue and adipocytes have been reported in previous studies as well. Adipocytes release adipokines and inflammatory factors, including leptin and interleukin-10, which may attenuate adverse immunological responses and thus help improve survival from critical diseases [34]. Adiponectin, a peptide secreted by adipocytes, was also reported to play an important role in critical illness. It is recognized as an insulin sensitizer, anti-atherosclerotic agent, and anti-inflammatory agent. Lower adiponectin levels on admission, followed by gradual elevation, may be a useful signal for a better prognosis of critical illness [35]. There is also evidence that patients with obesity can supply more lipoproteins, which can bind to endotoxins and lessen their toxic actions [36]. Additionally, tumor necrosis factor-alpha receptor has been observed to be upregulated in adipose tissue, which may aid in the dispersal of cardiomyocyte-activated arrhythmogenic substrates and inflammation [37]. Finally, obese patients have lower levels of natriuretic peptide, which has been shown to predict stroke and death among AF patients [38].

Other potential mechanisms included the fact that obese patients receive more attention from clinical staff and differences in baseline characteristics across BMI categories. Whether the obesity paradox really exists or is the result of selection bias has been hotly debated. Obese patients are often considered to have an increased risk of mortality and complications than normal-weight patients, which may result in earlier ICU admissions and more aggressive treatments [39, 40]. Interestingly, in our study population, overweight and obese patients received more relevant treatments, including MV, RRT, vasopressors, and antiplatelet agents. This is in line with the findings of these previous studies. Use of MV, RRT, and vasopressors are important organ support therapies in critically ill patients. Antiplatelet agents are regularly used for the prevention of stroke and thrombotic events in patients with atrial fibrillation [41, 42]. These aggressive treatments may be one of the reasons for the reduced risk of death in obese patients. Additionally, previous studies have shown that obese and overweight patients in the ICU tend to be younger, and our study has the same results [24, 43]. In our study, we observed a higher prevalence of hypertension and diabetes among overweight and obese patients, which may result in an earlier onset of AF [44]. Age is a significant predictor for stroke and mortality [45]; consequently, obese patients with AF may have better outcomes due to their younger age. In addition, it seems logical that, in our data, obese patients had a lower proportion of comorbid strokes in comparison to normal-weight patients. It has also been shown that less cachexia is related to the obesity paradox [46]. As a result, it is not surprising that underweight patients presented more history of malignancy in our cohort. Notwithstanding adjustments for age and other confounders in the multivariable regression models, this may not fully account for the differences in baseline characteristics across BMI categories. The findings should therefore be interpreted with caution.

Lower sympathetic activity in obese patients may also be involved in the mechanisms of the obesity paradox [47]. Studies have demonstrated that sympathetic overdrive plays a significant role in the progression of AF and is associated with poorer outcomes among AF patients [48]. Hence, lower activation of the sympathetic nervous system may have a protective effect in obese patients. Another potential mechanism focused on the genetic factors. Lean patients with cardiovascular disease (CVD) may develop CVD because of their completely different etiologies and genetic predispositions, which may be associated with poorer outcomes [49].

The current study has several limitations. First, data from the MIMIC-IV database did not include long-term mortality over one year or cardiovascular mortality, which limited our further analysis of the association between obesity and outcomes in critically ill patients with AF. Second, due to the complexity of ICD codes, errors may occur when they are used by medical staff. Therefore, identifying AF patients by ICD codes might not be the most accurate method. Unfortunately, we were unable to find a more accurate way to identify AF patients because of the nature of the MIMIC-IV database. Additionally, BMI is unable to quantify body fat percentage or distribution because it represents the sum of fat-free mass index and fat-mass index [50]. As in many previous studies, the definition of obesity in our study was based on BMI, which does not take into account different obesity phenotypes. Recent studies have suggested that various obesity phenotypes with different cardiovascular risk profiles coexist within the same BMI category [51]. When markers of central obesity, such as waist circumference or waist-hip ratio, are used instead of BMI, the opposite results may occur. Hence, to overcome the limitations of the traditional definition of obesity, a new classification of obesity based on different variables needs to be established in future studies. Finally, although our study from the MIMIC-IV database has a large sample size, there were only a small number of participants in the underweight category, which may affect the statistical power and reliability of our data analyses. Despite the above limitations, this study provides valuable information on the association between BMI and prognoses among critically ill patients with AF.

## Conclusions

This study revealed a nonlinear relationship between BMI and short- and medium-term all-cause mortality among critically ill patients with AF. All-cause mortality and the BMI were negatively correlated when the BMI was less than 30 kg/m^2^. These findings suggested that the obesity paradox may also be suitable for critically ill patients with AF. The mechanisms underlying this relationship were worthy of further investigation.

## Electronic supplementary material

Below is the link to the electronic supplementary material.

Supplementary Material 1

## Data Availability

All the data used to support this study are available from the corresponding author upon request.

## Abbreviations

AF: Atrial fibrillation
MIMIC-IV: Medical Information Mart for Intensive Care-IV
BMI: Body mass index
HR: Hazard ratio
CI: Confidence interval
ICU: Intensive care unit
BIDMC: Beth Israel Deaconess Medical Center
ICD: International Classification of Diseases
CHF: Congestive heart failure
PVD: Peripheral vascular disease
CPD: Chronic pulmonary disease
AKI: Acute kidney injury
AHF: Acute heart failure
SOFA: Sequential organ failure assessment
SAPS II: Simplified acute physiology score II
CCI: Charlson comorbidity index
WBC: White blood cell
RDW: Red cell distribution width
BUN: Blood urea nitrogen
HR: Heart rate
MBP: Mean blood pressure
RR: Respiratory rate
SpO_2_: Saturation of pulse oximetry
MV: Mechanical ventilation
RRT: Renal replacement therapy
SD: Standard deviation
IQR: Interquartile range
VIF: Variance inflation factor
CVD: Cardiovascular disease
