# Supplementary material for: Association between obesity and short- and medium-term mortality in critically ill patients with atrial fibrillation: a retrospective cohort study
Source: BMC Cardiovasc Disord. 2023 Mar 23;23:150. doi: 10.1186/s12872-023-03179-x (PMC10037857; doi:10.1186/s12872-023-03179-x)
Supplement: Supplementary file 1 — Supplementary Material 1 [file 12872_2023_3179_MOESM1_ESM.docx]

**Supplementary Table 1** Single-factor Cox regression analysis of covariates and all-cause mortality

| Variables | 30-day all-cause mortality | | 90-day all-cause mortality | | 1-year all-cause mortality | |
| --- | --- | --- | --- | --- | --- | --- |
|  | HR (95% CI) | *P*-Value | HR (95% CI) | *P*-Value | HR (95% CI) | *P*-Value |
| Age (years) | 1.04 (1.03, 1.05) | < 0.001 | 1.04 (1.04, 1.05) | < 0.001 | 1.04 (1.04, 1.04) | < 0.001 |
| Sex |  |  |  |  |  |  |
| Female | 1.0 |  | 1.0 |  | 1.0 |  |
| Male | 0.79 (0.71, 0.88) | < 0.001 | 0.79 (0.72, 0.86) | < 0.001 | 0.78 (0.72, 0.84) | < 0.001 |
| Race |  |  |  |  |  |  |
| Other | 1.0 |  | 1.0 |  | 1.0 |  |
| White | 0.76 (0.67, 0.85) | < 0.001 | 0.8 (0.73, 0.88) | < 0.001 | 0.94 (0.86, 1.02) | 0.124 |
| Hypertension |  |  |  |  |  |  |
| No | 1.0 |  | 1.0 |  | 1.0 |  |
| Yes | 0.67 (0.61, 0.75) | < 0.001 | 0.61 (0.56, 0.68) | < 0.001 | 0.59 (0.54, 0.64) | < 0.001 |
| Diabetes |  |  |  |  |  |  |
| No | 1.0 |  | 1.0 |  | 1.0 |  |
| Yes | 0.96 (0.86, 1.07) | 0.461 | 1.02 (0.92, 1.12) | 0.743 | 1.11 (1.02, 1.2) | 0.013 |
| CHF |  |  |  |  |  |  |
| No | 1.0 |  | 1.0 |  | 1.0 |  |
| Yes | 1.39 (1.25, 1.54) | < 0.001 | 1.48 (1.35, 1.62) | < 0.001 | 1.57 (1.46, 1.7) | < 0.001 |
| PVD |  |  |  |  |  |  |
| No | 1.0 |  | 1.0 |  | 1.0 |  |
| Yes | 1.05 (0.91, 1.21) | 0.476 | 1.11 (0.99, 1.25) | 0.078 | 1.17 (1.06, 1.29) | 0.001 |
| Cerebrovascular disease |  |  |  |  |  |  |
| No | 1.0 |  | 1.0 |  | 1.0 |  |
| Yes | 1.63 (1.44, 1.84) | < 0.001 | 1.51 (1.35, 1.69) | < 0.001 | 1.42 (1.3, 1.56) | < 0.001 |
| CPD |  |  |  |  |  |  |
| No | 1.0 |  | 1.0 |  | 1.0 |  |
| Yes | 1.04 (0.93, 1.16) | 0.502 | 1.08 (0.98, 1.19) | 0.108 | 1.16 (1.07, 1.25) | < 0.001 |
| Renal disease |  |  |  |  |  |  |
| No | 1.0 |  | 1.0 |  | 1.0 |  |
| Yes | 1.56 (1.4, 1.74) | < 0.001 | 1.67 (1.52, 1.83) | < 0.001 | 1.76 (1.63, 1.9) | < 0.001 |
| Liver disease |  |  |  |  |  |  |
| No | 1.0 |  | 1.0 |  | 1.0 |  |
| Yes | 2.48 (2.15, 2.85) | < 0.001 | 2.42 (2.14, 2.74) | < 0.001 | 2.16 (1.93, 2.41) | < 0.001 |
| Malignancy |  |  |  |  |  |  |
| No | 1.0 |  | 1.0 |  | 1.0 |  |
| Yes | 1.98 (1.74, 2.25) | < 0.001 | 2.15 (1.93, 2.4) | < 0.001 | 2.38 (2.17, 2.61) | < 0.001 |
| Sepsis |  |  |  |  |  |  |
| No | 1.0 |  | 1.0 |  | 1.0 |  |
| Yes | 2.49 (2.2, 2.82) | < 0.001 | 2.23 (2.01, 2.48) | < 0.001 | 1.83 (1.68, 1.98) | < 0.001 |
| AHF |  |  |  |  |  |  |
| No | 1.0 |  | 1.0 |  | 1.0 |  |
| Yes | 1.33 (1.18, 1.49) | < 0.001 | 1.42 (1.29, 1.57) | < 0.001 | 1.53 (1.41, 1.66) | < 0.001 |
| Stroke |  |  |  |  |  |  |
| No | 1.0 |  | 1.0 |  | 1.0 |  |
| Yes | 2.17 (1.89, 2.51) | < 0.001 | 2.02 (1.78, 2.29) | < 0.001 | 1.84 (1.64, 2.05) | < 0.001 |
| AKI |  |  |  |  |  |  |
| No | 1.0 |  | 1.0 |  | 1.0 |  |
| Yes | 3.13 (2.57, 3.81) | < 0.001 | 2.6 (2.23, 3.04) | < 0.001 | 1.8 (1.61, 2.01) | < 0.001 |
| SOFA | 1.18 (1.17, 1.19) | < 0.001 | 1.15 (1.14, 1.17) | < 0.001 | 1.12 (1.11, 1.13) | < 0.001 |
| SAPS Ⅱ | 1.05 (1.05, 1.06) | < 0.001 | 1.05 (1.04, 1.05) | < 0.001 | 1.04 (1.04, 1.04) | < 0.001 |
| CCI | 1.22 (1.19, 1.24) | < 0.001 | 1.23 (1.21, 1.25) | < 0.001 | 1.25 (1.23, 1.26) | < 0.001 |
| Hemoglobin (g/dL) | 1.02 (1, 1.04) | 0.087 | 0.9989 (0.9798, 1.0184) | 0.912 | 0.9908 (0.9749, 1.007) | 0.265 |
| Platelets (×10^9^/L) | 1.0015 (1.0011, 1.0019) | < 0.001 | 1.0016 (1.0012, 1.0019) | < 0.001 | 1.0016 (1.0013, 1.0019) | < 0.001 |
| WBC (×10^9^/L) | 1.0094 (1.0069, 1.0118) | < 0.001 | 1.0085 (1.0061, 1.0109) | < 0.001 | 1.0071 (1.0046, 1.0095) | < 0.001 |
| RDW (%) | 1.19 (1.17, 1.2) | < 0.001 | 1.19 (1.17, 1.21) | < 0.001 | 1.2 (1.18, 1.21) | < 0.001 |
| Anion gap (mmol/L) | 1.1 (1.09, 1.11) | < 0.001 | 1.09 (1.09, 1.1) | < 0.001 | 1.09 (1.08, 1.09) | < 0.001 |
| BUN (mg/dL) | 1.01 (1.01, 1.02) | < 0.001 | 1.01 (1.01, 1.02) | < 0.001 | 1.01 (1.01, 1.02) | < 0.001 |
| Creatinine (mg/dL) | 1.15 (1.12, 1.18) | < 0.001 | 1.14 (1.12, 1.17) | < 0.001 | 1.15 (1.13, 1.16) | < 0.001 |
| Glucose (mg/dL) | 1.0015 (1.0011, 1.0018) | < 0.001 | 1.0014 (1.0011, 1.0017) | < 0.001 | 1.0013 (1.001, 1.0016) | < 0.001 |
| HR (beats/minute) | 1.01 (1.01, 1.01) | < 0.001 | 1.01 (1.01, 1.01) | < 0.001 | 1.01 (1.01, 1.01) | < 0.001 |
| MBP (mmHg) | 0.9982 (0.9953,1.0012) | 0.238 | 0.9972 (0.9947, 0.9998) | 0.035 | 0.9982 (0.996, 1.0003) | 0.098 |
| RR (beats/minute) | 1.06 (1.05, 1.07) | < 0.001 | 1.06 (1.05, 1.07) | < 0.001 | 1.06 (1.05, 1.06) | < 0.001 |
| SpO_2_ (%) | 0.95 (0.95, 0.96) | < 0.001 | 0.96 (0.95, 0.96) | < 0.001 | 0.96 (0.95, 0.96) | < 0.001 |
| Antiplatelet agents |  |  |  |  |  |  |
| No | 1.0 |  | 1.0 |  | 1.0 |  |
| Yes | 0.51 (0.45, 0.58) | < 0.001 | 0.57 (0.52, 0.64) | < 0.001 | 0.62 (0.57, 0.68) | < 0.001 |
| Anticoagulant agents |  |  |  |  |  |  |
| No | 1.0 |  | 1.0 |  | 1.0 |  |
| Yes | 0.81 (0.73, 0.9) | < 0.001 | 0.89 (0.82, 0.98) | 0.013 | 0.96 (0.89, 1.03) | 0.282 |
| Antiarrhythmic agents |  |  |  |  |  |  |
| No | 1.0 |  | 1.0 |  | 1.0 |  |
| Yes | 0.72 (0.65, 0.8) | < 0.001 | 0.78 (0.72, 0.86) | < 0.001 | 0.82 (0.76, 0.88) | < 0.001 |
| MV |  |  |  |  |  |  |
| No | 1.0 |  | 1.0 |  | 1.0 |  |
| Yes | 2.15 (1.93, 2.39) | < 0.001 | 1.85 (1.69, 2.02) | < 0.001 | 1.49 (1.38, 1.61) | < 0.001 |
| RRT |  |  |  |  |  |  |
| No | 1.0 |  | 1.0 |  | 1.0 |  |
| Yes | 2.88 (2.5, 3.32) | < 0.001 | 2.79 (2.46, 3.16) | < 0.001 | 2.59 (2.32, 2.9) | < 0.001 |
| Vasopressors |  |  |  |  |  |  |
| No | 1.0 |  | 1.0 |  | 1.0 |  |
| Yes | 1.39 (1.25, 1.55) | < 0.001 | 1.17 (1.07, 1.29) | < 0.001 | 0.99 (0.92, 1.07) | 0.762 |

Notes: data presented are HRs and 95% CIs.

Abbreviations: HR, hazard ratio; CI, confidence interval; CHF, congestive heart failure; PVD, peripheral vascular disease; CPD, chronic pulmonary disease; AKI, acute kidney injury; AHF, acute heart failure; SOFA, sequential organ failure assessment; SAPS Ⅱ, simplified acute physiology score Ⅱ; CCI, Charlson comorbidity index; WBC, white blood cell; RDW, red cell distribution width; BUN, blood urea nitrogen; HR, heart rate; MBP, mean blood pressure; RR, respiratory rate; SpO_2_, saturation of pulse oximetry; MV, mechanical ventilation; RRT, renal replacement therapy.

**Supplementary Table 2** Relationship between BMI and all-cause mortality after excluding participants with incomplete data of covariates

| Variables | Crude model | | Model 1 | | Model 2 | |
| --- | --- | --- | --- | --- | --- | --- |
|  | HR (95% CI) | *P*-value | HR (95% CI) | *P*-value | HR (95% CI) | *P*-value |
| 30-day all-cause mortality |  |  |  |  |  |  |
| BMI category |  |  |  |  |  |  |
| Underweight | 1.93 (1.49, 2.5) | <0.001 | 1.96 (1.51, 2.54) | <0.001 | 1.62 (1.24, 2.12) | <0.001 |
| Normal-weight | 1.00 (Reference) |  | 1.00 (Reference) |  | 1.00 (Reference) |  |
| Overweight | 0.76 (0.66, 0.86) | <0.001 | 0.85 (0.75, 0.97) | 0.018 | 0.83 (0.72, 0.94) | 0.005 |
| Obese | 0.61 (0.53, 0.69) | <0.001 | 0.79 (0.69, 0.91) | 0.001 | 0.8 (0.69, 0.92) | 0.002 |
| *P* for trend |  | <0.001 |  | <0.001 |  | <0.001 |
| 90-day all-cause mortality |  |  |  |  |  |  |
| BMI category |  |  |  |  |  |  |
| Underweight | 1.73 (1.37, 2.17) | <0.001 | 1.76 (1.4, 2.22) | <0.001 | 1.48 (1.17, 1.88) | 0.001 |
| Normal-weight | 1.00 (Reference) |  | 1.00 (Reference) |  | 1.00 (Reference) |  |
| Overweight | 0.7 (0.63, 0.79) | <0.001 | 0.79 (0.71, 0.88) | <0.001 | 0.77 (0.68, 0.86) | <0.001 |
| Obese | 0.55 (0.49, 0.62) | <0.001 | 0.72 (0.64, 0.81) | <0.001 | 0.71 (0.63, 0.8) | <0.001 |
| *P* for trend |  | <0.001 |  | <0.001 |  | <0.001 |
| 1-year all-cause mortality |  |  |  |  |  |  |
| BMI category |  |  |  |  |  |  |
| Underweight | 1.72 (1.41, 2.1) | <0.001 | 1.75 (1.43, 2.13) | <0.001 | 1.48 (1.21, 1.82) | <0.001 |
| Normal-weight | 1.00 (Reference) |  | 1.00 (Reference) |  | 1.00 (Reference) |  |
| Overweight | 0.7 (0.64, 0.77) | <0.001 | 0.78 (0.71, 0.86) | <0.001 | 0.76 (0.7, 0.84) | <0.001 |
| Obese | 0.56 (0.51, 0.62) | <0.001 | 0.72 (0.66, 0.8) | <0.001 | 0.7 (0.63, 0.78) | <0.001 |
| *P* for trend |  | <0.001 |  | <0.001 |  | <0.001 |

Note: The sample size was 9111. Crude model was adjusted for none; Model 1 was adjusted for age, sex and race; Model 2 was further adjusted (from Model 1) for hypertension, diabetes, CHF, PVD, cerebrovascular disease, CPD, renal disease, liver disease, malignancy, sepsis, AKI, AHF, stroke, SOFA, SAPS Ⅱ, CCI, hemoglobin, WBC, platelet, RDW, anion gap, BUN, creatinine, glucose, HR, MBP, RR, SpO_2_, antiplatelet agents, anticoagulant agents, antiarrhythmic agents, MV, RRT, and vasopressors.

Abbreviations: BMI, body mass index; HR, hazard ratio; CI, confidence interval; CHF, congestive heart failure; PVD, peripheral vascular disease; CPD, chronic pulmonary disease; AKI, acute kidney injury; AHF, acute heart failure; SOFA, sequential organ failure assessment; SAPS Ⅱ, simplified acute physiology score Ⅱ; CCI, Charlson comorbidity index; WBC, white blood cell; RDW, red cell distribution width; BUN, blood urea nitrogen; HR, heart rate; MBP, mean blood pressure; RR, respiratory rate; SpO_2_, saturation of pulse oximetry; MV, mechanical ventilation; RRT, renal replacement therapy.

**Supplementary** **Table 3** Threshold effect analysis of the relationship between BMI and all-cause mortality after excluding participants with incomplete data of covariates

| Threshold of BMI |  | 30-day all-cause mortality | |  | 90-day all-cause mortality | |  | 1-year all-cause mortality | |
| --- | --- | --- | --- | --- | --- | --- | --- | --- | --- |
|  |  | HR (95%CI) | *P*-value |  | HR (95%CI) | *P*-value |  | HR (95%CI) | *P*-value |
| <30kg/m^2^ |  | 0.937 (0.919, 0.955) | <0.001 |  | 0.931 (0.915, 0.946) | <0.001 |  | 0.932 (0.919, 0.946) | <0.001 |
| ≥30kg/m^2^ |  | 0.995 (0.974, 1.017) | 0.663 |  | 0.992 (0.974, 1.011) | 0.417 |  | 0.996 (0.981, 1.012) | 0.62 |
| Likelihood ratio test |  |  | <0.001 |  |  | <0.001 |  |  | <0.001 |

Note: HRs were adjusted for age, sex, race, hypertension, diabetes, CHF, PVD, cerebrovascular disease, CPD, renal disease, liver disease, malignancy, sepsis, AKI, AHF, stroke, SOFA, SAPS Ⅱ, CCI, hemoglobin, WBC, platelet, RDW, anion gap, BUN, creatinine, glucose, HR, MBP, RR, SpO_2_, antiplatelet agents, anticoagulant agents, antiarrhythmic agents, MV, RRT, and vasopressors.

Abbreviations: BMI, body mass index; HR, hazard ratio; CI, confidence interval; CHF, congestive heart failure; PVD, peripheral vascular disease; CPD, chronic pulmonary disease; AKI, acute kidney injury; AHF, acute heart failure; SOFA, sequential organ failure assessment; SAPS Ⅱ, simplified acute physiology score Ⅱ; CCI, Charlson comorbidity index; WBC, white blood cell; RDW, red cell distribution width; BUN, blood urea nitrogen; HR, heart rate; MBP, mean blood pressure; RR, respiratory rate; SpO_2_, saturation of pulse oximetry; MV, mechanical ventilation; RRT, renal replacement therapy.

**Supplementary Table 4** Relationship between BMI and all-cause mortality when patients with BMI ≥ 30 kg/m^2^ are divided into obese group and morbidly obese group

| Variables | Crude model | | Model 1 | | Model 2 | |
| --- | --- | --- | --- | --- | --- | --- |
|  | HR (95% CI) | *P*-value | HR (95% CI) | *P*-value | HR (95% CI) | *P*-value |
| 30-day all-cause mortality |  |  |  |  |  |  |
| BMI category |  |  |  |  |  |  |
| Underweight | 1.89 (1.46, 2.44) | <0.001 | 1.92 (1.48, 2.48) | <0.001 | 1.58 (1.21, 2.07) | 0.001 |
| Normal-weight | 1.00 (Reference) |  | 1.00 (Reference) |  | 1.00 (Reference) |  |
| Overweight | 0.75 (0.66, 0.85) | <0.001 | 0.84 (0.74, 0.96) | 0.011 | 0.82 (0.72, 0.93) | 0.003 |
| Obese | 0.6 (0.52, 0.69) | <0.001 | 0.77 (0.66, 0.89) | <0.001 | 0.79 (0.68, 0.92) | 0.002 |
| Morbidly obese | 0.59 (0.46, 0.74) | <0.001 | 0.87 (0.69, 1.11) | 0.272 | 0.77 (0.6, 0.98) | 0.034 |
| *P* for trend |  | <0.001 |  | <0.001 |  | <0.001 |
| 90-day all-cause mortality |  |  |  |  |  |  |
| BMI category |  |  |  |  |  |  |
| Underweight | 1.7 (1.35, 2.14) | <0.001 | 1.73 (1.38, 2.18) | <0.001 | 1.46 (1.15, 1.85) | 0.002 |
| Normal-weight | 1.00 (Reference) |  | 1.00 (Reference) |  | 1.00 (Reference) |  |
| Overweight | 0.7 (0.63, 0.78) | <0.001 | 0.79 (0.7, 0.88) | <0.001 | 0.76 (0.68, 0.85) | <0.001 |
| Obese | 0.55 (0.49, 0.62) | <0.001 | 0.7 (0.62, 0.79) | <0.001 | 0.71 (0.62, 0.8) | <0.001 |
| Morbidly obese | 0.54 (0.44, 0.66) | <0.001 | 0.8 (0.65, 0.99) | 0.036 | 0.68 (0.55, 0.84) | <0.001 |
| *P* for trend |  | <0.001 |  | <0.001 |  | <0.001 |
| 1-year all-cause mortality |  |  |  |  |  |  |
| BMI category |  |  |  |  |  |  |
| Underweight | 1.69 (1.39, 2.06) | <0.001 | 1.72 (1.41, 2.09) | <0.001 | 1.45 (1.19, 1.78) | <0.001 |
| Normal-weight | 1.00 (Reference) |  | 1.00 (Reference) |  | 1.00 (Reference) |  |
| Overweight | 0.7 (0.63, 0.76) | <0.001 | 0.78 (0.71, 0.85) | <0.001 | 0.76 (0.69, 0.83) | <0.001 |
| Obese | 0.56 (0.5, 0.62) | <0.001 | 0.7 (0.63, 0.77) | <0.001 | 0.7 (0.62, 0.77) | <0.001 |
| Morbidly obese | 0.58 (0.49, 0.68) | <0.001 | 0.83 (0.7, 0.99) | 0.034 | 0.7 (0.59, 0.83) | <0.001 |
| *P* for trend |  | <0.001 |  | <0.001 |  | <0.001 |

Note: Crude model was adjusted for none; Model 1 was adjusted for age, sex and race; Model 2 was further adjusted (from Model 1) for hypertension, diabetes, CHF, PVD, cerebrovascular disease, CPD, renal disease, liver disease, malignancy, sepsis, AKI, AHF, stroke, SOFA, SAPS Ⅱ, CCI, hemoglobin, WBC, platelet, RDW, anion gap, BUN, creatinine, glucose, HR, MBP, RR, SpO_2_, antiplatelet agents, anticoagulant agents, antiarrhythmic agents, MV, RRT, and vasopressors.

Abbreviations: BMI, body mass index; HR, hazard ratio; CI, confidence interval; CHF, congestive heart failure; PVD, peripheral vascular disease; CPD, chronic pulmonary disease; AKI, acute kidney injury; AHF, acute heart failure; SOFA, sequential organ failure assessment; SAPS Ⅱ, simplified acute physiology score Ⅱ; CCI, Charlson comorbidity index; WBC, white blood cell; RDW, red cell distribution width; BUN, blood urea nitrogen; HR, heart rate; MBP, mean blood pressure; RR, respiratory rate; SpO_2_, saturation of pulse oximetry; MV, mechanical ventilation; RRT, renal replacement therapy.


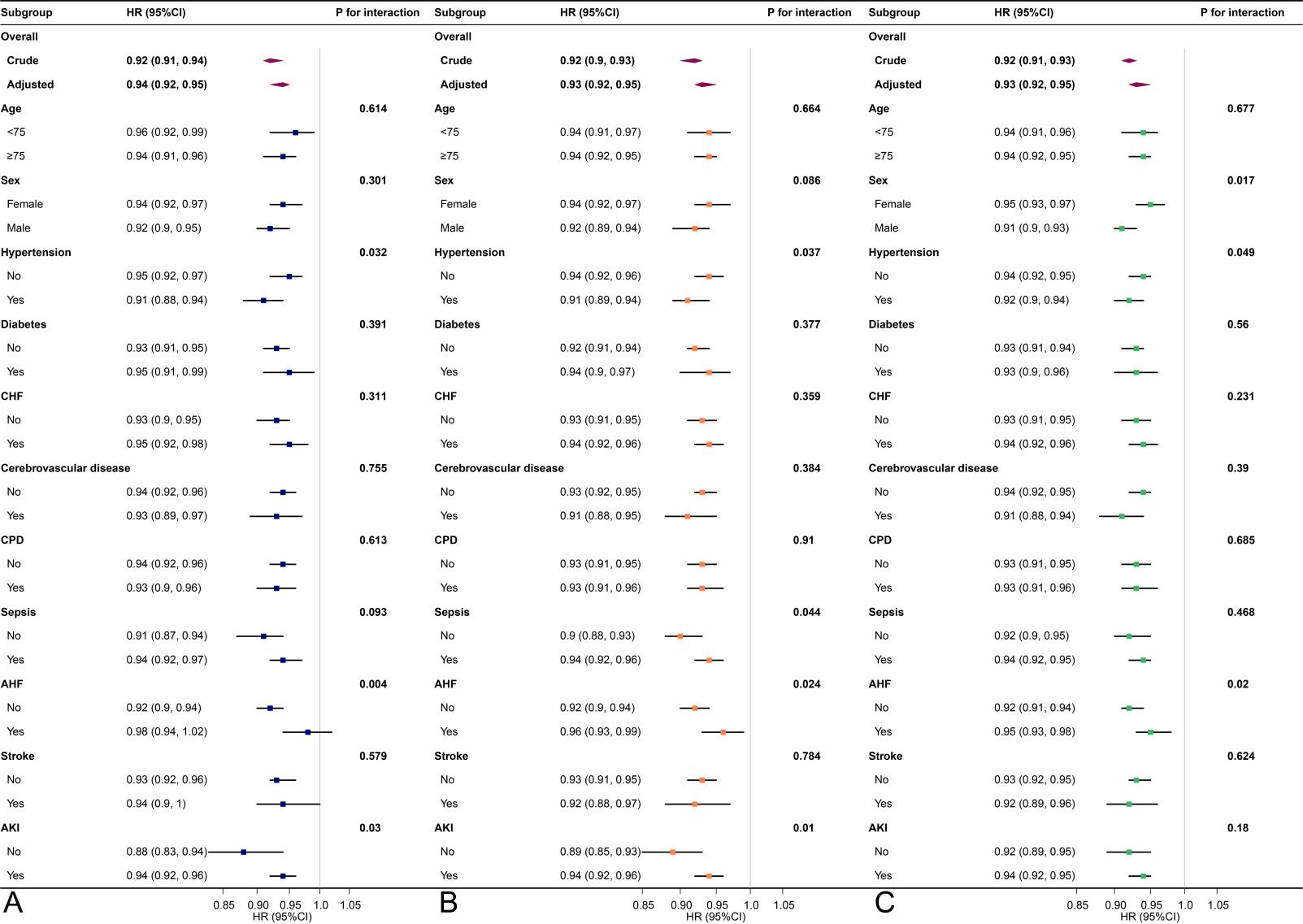


**Supplementary Fig. 1** Stratified analyses of patients with BMI < 30 kg/m^2^. The forest plot shows the results of subgroup analyses of 30-day (A), 90-day (B), and 1-year (C) mortality. HRs were adjusted for age, sex , race, hypertension, diabetes, CHF, PVD, cerebrovascular disease, CPD, renal disease, liver disease, malignancy, sepsis, AKI, AHF, stroke, SOFA, SAPS Ⅱ, CCI, hemoglobin, WBC, platelet, RDW, anion gap, BUN, creatinine, glucose, HR, MBP, RR, SpO_2_, antiplatelet agents, anticoagulant agents, antiarrhythmic agents, MV, RRT, and vasopressors.

Abbreviations: BMI, body mass index; HR, hazard ratio; CI, confidence interval; CHF, congestive heart failure; PVD, peripheral vascular disease; CPD, chronic pulmonary disease; AKI, acute kidney injury; AHF, acute heart failure; SOFA, sequential organ failure assessment; SAPS Ⅱ, simplified acute physiology score Ⅱ; CCI, Charlson comorbidity index; WBC, white blood cell; RDW, red cell distribution width; BUN, blood urea nitrogen; HR, heart rate; MBP, mean blood pressure; RR, respiratory rate; SpO_2_, saturation of pulse oximetry; MV, mechanical ventilation; RRT, renal replacement therapy.


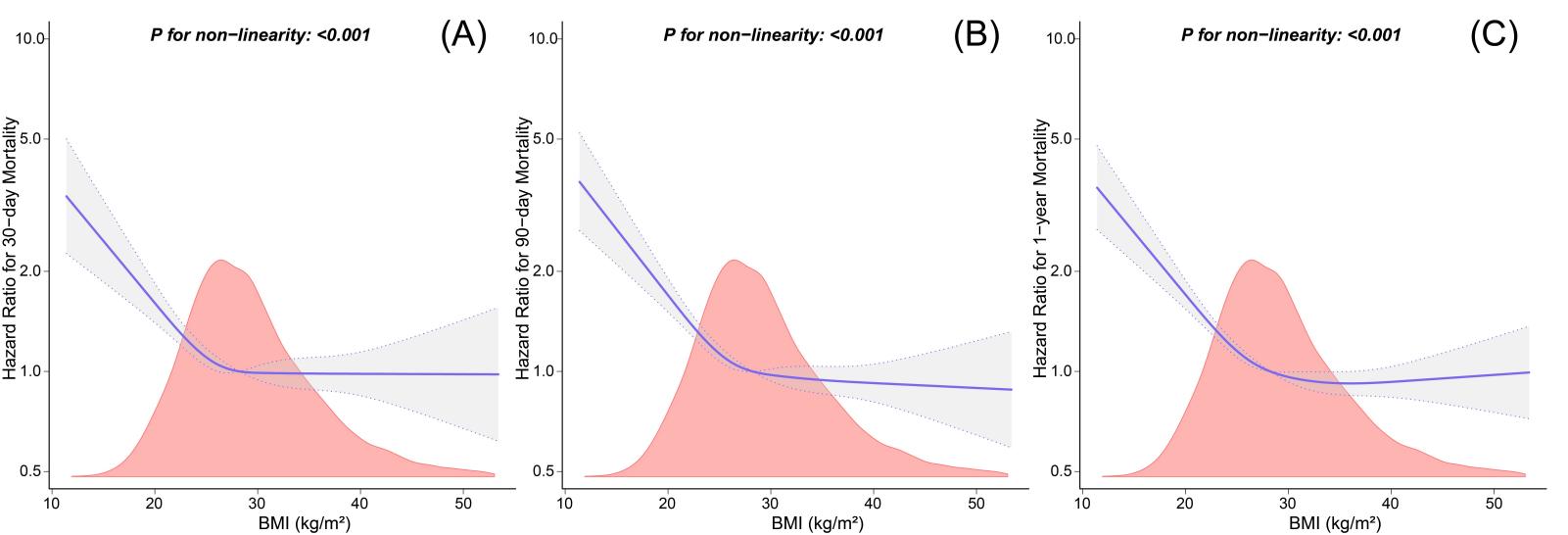


**Supplementary Fig. 2** Multivariable-adjusted restricted cubic spline analyses of relationship between BMI and all-cause mortality using complete dataset. The upper limit of the BMI is restricted to 99th. The purple lines represent the estimated risk of all-cause mortality, and the gray bands represent the point-by-point 95% CI adjusted for covariates. HRs were adjusted for age, sex , race, hypertension, diabetes, CHF, PVD, cerebrovascular disease, CPD, renal disease, liver disease, malignancy, sepsis, AKI, AHF, stroke, SOFA, SAPS Ⅱ, CCI, hemoglobin, WBC, platelet, RDW, anion gap, BUN, creatinine, glucose, HR, MBP, RR, SpO_2_, antiplatelet agents, anticoagulant agents, antiarrhythmic agents, MV, RRT, and vasopressors.

Abbreviations: BMI, body mass index; HR, hazard ratio; CI, confidence interval; CHF, congestive heart failure; PVD, peripheral vascular disease; CPD, chronic pulmonary disease; AKI, acute kidney injury; AHF, acute heart failure; SOFA, sequential organ failure assessment; SAPS Ⅱ, simplified acute physiology score Ⅱ; CCI, Charlson comorbidity index; WBC, white blood cell; RDW, red cell distribution width; BUN, blood urea nitrogen; HR, heart rate; MBP, mean blood pressure; RR, respiratory rate; SpO_2_, saturation of pulse oximetry; MV, mechanical ventilation; RRT, renal replacement therapy.
